# A Protestation

**Published:** 1895-10

**Authors:** A. Liautard

**Affiliations:** Paris


### A PROTESTATION.

PROF. OLOF SCHWARZKOPF, V.M.D., *Chairman of Executive Committee, Association of Veterinary Faculties of North America:*

DEAR SIR: I have received your letter of July 13th, with the programme of the next meeting of the "self-named Association of Veterinary Faculties of North America," doing me the high honor of "either approving, altering, or suggesting, as I may see fit." I thank you for the compliment, and though I know well that I am pleading for a lost cause, and that it is simply . . . foolish for me to hope that my efforts may carry their point, notwithstanding these expectations, I will permit myself to take advantage of your invitation, not to approve, alter, or suggest the programme, but simply to call *your* attention, that of the *members* of the Association of the Veterinary Faculties of North America, and, above all, that of *every* member of the United States Veterinary Medical Association who has at heart the name of the Association, her reputation, and her welfare, to only one point—that is, to the UNCONSTITUTIONALITY OF YOUR ACTIONS, and if I am not going to approve, alter, or suggest on the programme; what I intend to do is to ask you to stop where you are, Mr. Chairman, and to tell you and your colleagues of the Association of the Veterinary Faculties of North America:

*Stop!* or, rather, *begin over again!*

Yes, *stop*, because you are illegal!

Yes, *begin over again*, because the work that you may do, the subjects that you may consider, the laws or regulations that you may enact, will all be thrown away uselessly, because it cannot be entertained; because, remaining a dead letter, it will do no good to the noble work you intend to do, no good to the great reform that you will attempt to bring out; because in this land of liberty arbitral actions are illegal; and because, if your existence may serve personalities, it will do no good to American veterinary medicine.

What you may say is: "Is it Dr. Liautard who speaks thus?" "Is it he, who, after being the leading advocate of the very subjects of the programme, now asks to drop them?" "Is it he, who, at the first Veterinary Congress of America, expected what he had on previous occasions urged in his paper on 'Veterinary Education: As It Was, As It Is, and As It Should be?'" "Is it he, who, before the New York State Veterinary Medical Society, presented an address to the same effect?" "Is it he who tells us to stop when we are about considering the reforms which he has so urgently asked of us, and which, should they be brought to effect, would crown his professional work in behalf of veterinary medicine with the most glorious reward?"

Yes, it is I! and why?

Mr. Chairman, let me ask a single question: *By what authority does the self-named Association of Veterinary Faculties exist?*

It may be answered by direction of that great Association for which we all work, by that great body of veterinarians, who for years have endeavored to elevate the profession, and have succeeded. Right! I grant that it became a duty for the United States Veterinary Medical Association to ask the formation of the Association of Veterinary Faculties of North America, which I have myself asked for, and which I ask for again.

But—and here are my objections to her existence, to her right of existence, to her legality—and, if I am right, I say and I repeat: *Stop where you are and begin over again.*

That which I am now going to say I wanted to say last year at the meeting at Philadelphia. Landed but just a few hours from a trip across the Atlantic, and finding my inability to be at the meeting before action was taken on the report and discussion on the new-born society, I had telegraphed to my friend, Prof. Robertson, to ask for a short recess, a brief delay, to allow me to arrive from New York. This request was denied, and I just entered the room to hear that the child was born—*a child doomed to death from its birth.* What I wanted to say at the meeting I told you, Mr. Chairman, in Philadelphia, and my remarks at that time you seemed to approve.

What were these remarks? They were imbedded in the following words, or about, viz.: "*That the Association of Veterinary Faculties of North America was not properly organized; that the fact of a* FEW *persons engaged in teaching in* SOME *of the veterinary colleges of North America being asked to unite for the purpose of organizing had no value; that the only proper way to have the orgenization possessed of a solid standing was to have the* OFFICERS *of the colleges notified of the project in view. and they to delegate professors from their faculties to represent each individual college.* Speaking of *officers* of a college, I do not mean the teachers, the professors, nor the dean, nor the principal. I mean *the board of directors*, the *board of trustees*, the GUARDIANS of the charter, which is the life of a school.

WAS THIS DONE? If it was not, I say it again, *Stop and begin over again!*

You know, Mr. Chairman, it was not done.

Instead of that which, in my humble estimation, ought to have been done, what did take place?

With, perhaps, some slight errors of no value, I know it was thus: NOTICES were sent to SOME, to very few, not to all, *not to every member of* SOME faculties.

Was this correct? Why to a few only? Were these few the master of all? Why should they be invited to assume certain powers, when only those who had that power did not delegate them to assume it?

These notices mentioned the intention to have a meeting to organize at the annual gathering at Philadelphia. Some were present, others were absent (I, unfortunately, among those). The organization took place, officers were elected, and as I said, the *child was born in a hurry, a premature delivery.* Afterwards notices were sent to those who had been elected members of the Association; they were placed on various committees, and to-day you prepare, yourself, the second act of that which, should not the subject be so important, and should I not fear to indulge in personalties, I would call *a farce, a comedy.*

Of what value can be the decision resulting from the discussion of the subjects on "Prescribed Entrance Examination;" on "State Boards of Veterinary Examiners and their Relations to Veterinary Colleges;" on "Uniform Degrees;" on "Competitive Examinations for Veterinary Faculties," with an amalgam like that which composes your organization, viz.:

1st. *Representatives, mostly all, without sanction, authority, or power to represent.*

2d. *Representatives of two- and three-year schools.*

3d. *Representatives of private institutions and of colleges attached to universities as departments;* and

4th. In the presence of schools which exist now and are not represented in your Association of *faculties*, and, still more, who are not represented in the mother national body.

Mr. Chairman, the work that lays before you seems to me very simple, and can be performed at once, without any one fearing that his pride has lost any of its right. *You recognize your illegal organization, and state so to the Association.* You ask power to continue your work as a committee, not as an association, and then *begin over again* in obtaining your OFFICIAL appointment from the trustees and directors of the various places where veterinary education is carried out. You can then reorganize later on, and be prepared to enter into the performance of your work "*as it should be*," and with the certainty of receiving the approval and support of every member of the profession.

Mr. Chairman, I am miles away from Des Moines. On account of unavoidable circumstances I am once more unable to be at the meeting of the National Society, and must trust to writing that which I fear can scarcely be communicated, as it should be, except by *one fully convinced.* Have I succeeded in giving you, or any of your colleagues, *that conviction?* I hope I have. And, if such is the case, I shall be grateful to all of you to have listened, not to my words, but to the laws of rightfulness and equity; and, if you permit me, I will then renew my efforts, offer my services for the cause in question—the elevation, the perfecting of education in *American veterinary institutions.*

Yours truly,

A. LIAUTARD, M.D., V.M.

PARIS, August 1, 1895.

